# Estrous cycle and ovariectomy-induced changes in visceral pain are microbiota-dependent

**DOI:** 10.1016/j.isci.2021.102850

**Published:** 2021-07-10

**Authors:** Mónica Tramullas, James M. Collins, Patrick Fitzgerald, Timothy G. Dinan, Siobhain M. O’ Mahony, John F. Cryan

**Affiliations:** 1 APC Microbiome Ireland, University College Cork, Cork, Ireland; 2 Department of Anatomy and Neuroscience, University College Cork, Cork, Ireland; 3 Department of Psychiatry and Behavioural Science, University College Cork, Cork, Ireland

**Keywords:** Endocrinology, Microbiome, Neuroscience

## Abstract

Visceral hypersensitivity (VH) is a hallmark of many functional gastrointestinal disorders including irritable bowel syndrome and is categorized by a dull, diffuse sensation of abdominal pain. Recently, the gut microbiota has been implicated in VH in male mice, but the effects in females have yet to be explored fully. To this end, we now show that somewhat surprisingly, female germ-free mice have similar visceral pain responses to colorectal distension (CRD) as their conventional controls. However, we show that although sensitivity to CRD is estrous cycle stage-dependent in conventional mice, it is not in germ-free mice. Further, ovariectomy (OVX) induced VH in conventional but not germ-free mice, and induced weight gain regardless of microbiota status. Finally, we show that estrogen-replacement ameliorated OVX-induced VH. Taken together, this study provides evidence for a major role of female sex hormones and the gut microbiota in sensation of visceral pain in females.

## Highlights

• The visceral pain response is estrous cycle-dependent in conventional females only
• Ovariectomy induced visceral hypersensitivity in control but not germ-free females
• Ovariectomy-induced visceral hyperalgesia is ameliorated by estrogen replacement
• Novel findings on estrous cycle-dependent mechanisms of visceral pain

## Graphical abstract

**Figure:**
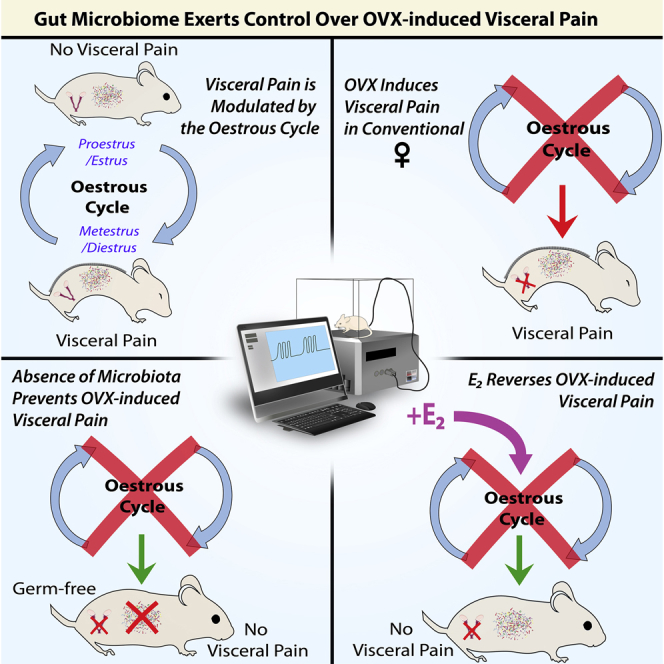

## Introduction

Visceral pain is a common and complex occurrence categorized by a diffuse, often dull sensation of pain centered around the midline and upper abdomen originating from some, but not all internal organs (Cervero and Laird, 1999; Sikandar and Dickenson, 2012). It has been reported that 25% of adults experience intermittent abdominal pain during their lifetime, highlighting the need for a better understanding of the pathophysiology of this disorder (Collett, 2013; Drewes et al., 2020). Visceral pain-associated disorders such as irritable bowel syndrome (IBS) are more commonly presented in women; however, to date the majority of preclinical studies on visceral pain are carried out exclusively in males, with their results being generalized to include females in terms of treatment of these disorders (Lee et al., 2018). This raises the issue of sex differences in treatment strategies and supports the notion that these treatments for disorders should not follow a singular approach across sexes.

The estrous cycle is the term used to describe the female reproductive cycle in rodents and is similar to the menstrual cycle in humans. In rodents, this cycle has four phases: (i) proestrus, (ii) estrus, (iii) metestrus, and (iv) diestrus, and lasts between 4 and 5 days (Byers et al., 2012). During this time, circulating gonadal hormone levels fluctuate. For example, levels of estrogen are highest during proestrus and lower in diestrus (Hong and Choi, 2018). Interestingly, it has been shown that the visceral pain response varies across the estrous cycle (Moloney et al., 2016). However, not all studies agree on the specific changes in visceral pain perception across the estrous cycle, with some reporting heightened visceral sensation when in proestrus versus metestrus/diestrus, or the converse (Ji et al., 2008; Giamberardino et al., 1997).

Ovariectomy (OVX) in rodents is used to cease the main production of female gonadal hormones (Lemini et al., 2015) and is a useful experimental model to investigate the specific effects of sex hormones on physiological parameters. Moreover, the effects of OVX on visceral pain processing are unclear as it has been shown that OVX results in a reduction in the visceromotor response (VMR) to colorectal distension (CRD) (Ji et al., 2003), or has no effect on visceral pain perception in rats (Traub et al., 2014). Here, we investigate the impact of OVX in female mice.

The gastrointestinal (GI) tract is home to between 10 and 100 trillion microbial cells which comprise the gut microbiota (Gilbert et al., 2018; Lynch and Pedersen, 2016; Blaser, 2014). The gut microbiota forms an essential component of the bidirectional communication between the gut and the nervous system, the microbiota-gut-brain axis, which has received increasingly more attention in recent years for its apparent role in the pathophysiology of many disorders of the gut-brain axis including IBS, a functional GI disorder characterized by visceral pain (Wilmes et al., 2021; Collins, 2014; Mayer et al., 2015; Bhattarai et al., 2016; Margolis et al., 2021).

There is also an increasing realization that signaling across the microbiota-gut brain axis is sex dependent (Jaggar et al., 2020; Jašarević et al., 2016). There is substantial evidence for the influence of gonadal hormones on the gut microbiota and vice versa (Tetel et al., 2018), with differences being observed between male and female rodents, as well as effects of gonadectomy and hormone replacement on the microbiota (Org et al., 2016; Jašarević et al., 2016). Altered levels of steroid hormones seen in germ-free (GF) mice, which are devoid of a microbiota and are sterile in microbiological terms (Spichak et al., 2018), further validates the interaction between the microbiota and gonadal hormones (Kamimura et al., 2019).

There is an increasing emphasis on the role of the microbiota in pain responses, particularly in visceral pain (Rea et al., 2017; Rea et al., 2019; O' Mahony et al., 2017; Luczynski et al., 2017; Theodorou et al., 2014; Defaye et al., 2020). We have previously shown that the gut microbiota plays a role in the mediation of visceral pain, whereby male GF mice displayed visceral hypersensitivity (VH) to CRD which was reduced following microbial colonization of the GI tract (Luczynski et al., 2017). Furthermore, it has also been shown that modulation of the gut microbiota in rodents by use of antibiotics in early life induces VH (O'Mahony et al., 2014). Moreover, manipulation of the gut microbiota using probiotic bacterial species including strains of *Bifidobacterium*, *Lactobacillus,* and *Streptococcus* (Dai et al., 2012; McKernan et al., 2010; Miquel et al., 2016; Verdú et al., 2006), as well as their soluble mediators (McVey Neufeld et al., 2020) has been shown to reduce the visceral pain response.

The underlying sex-dependent effects on visceral pain processing remain largely unexplored, particularly in the context of the gut microbiota. In this study, we investigated the role of the gut microbiota in the visceral pain response in female mice across the estrus cycle and in response to OVX.

## Results

### Absence of microbiota does not affect the visceral pain response to colorectal distension in female mice

To assess the role of the microbiota in the visceral pain response, the response to CRD in GF and conventionally colonized (CC) animals was investigated (see STAR Methods). To investigate the effect of cessation of production of circulating sex hormones, 6-week-old animals were ovariectomized or underwent sham surgery (see STAR Methods). These animals underwent CRD at 9 weeks of age.

**Figure 1:**
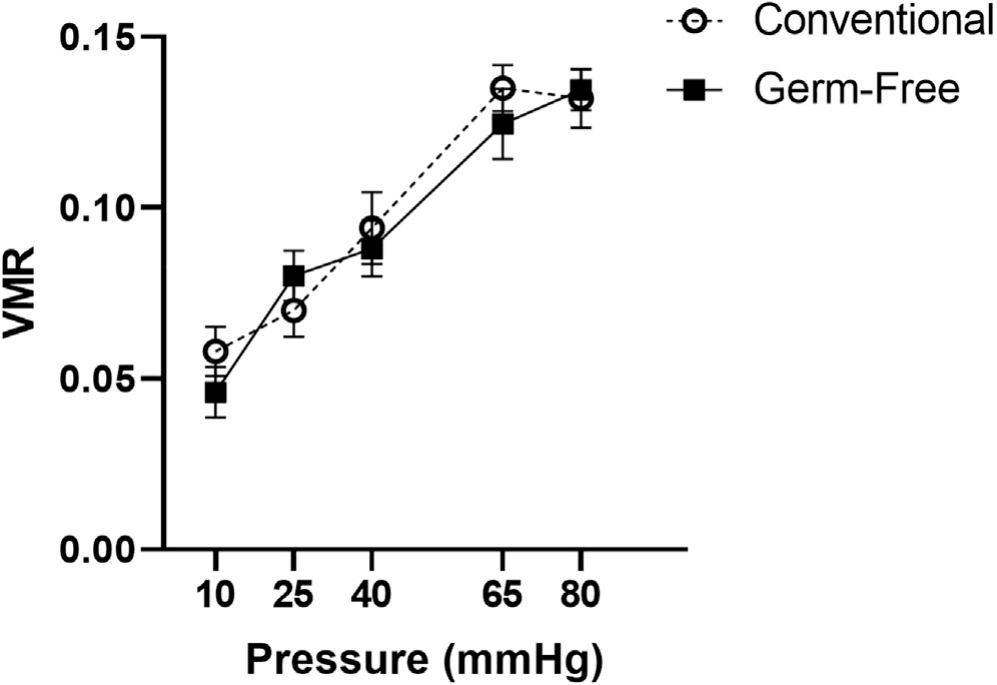
Absence of microbiota does not affect the visceral pain response to colorectal distension in female mice No differences observed in the visceromotor response (VMR) in response to colorectal distension between conventional and germ-free animals. Data presented as Mean ± SEM. n = 10 per group.

Interestingly, unlike what we have previously shown in male mice (Luczynski et al., 2017), a lack of microbiota did not affect the VMR to CRD in female mice. A mixed design ANOVA with pressure as the repeated measures factor and microbiota status as the independent factor showed a main effect of pressure (F(4,52) = 33.3, p<0.001) but not of microbiota status with respect to pressure (F(4,52) = 0.7, p=0.62) nor of microbiota status alone (F(1,13) = 0.8, p = 0.402) (Figure 1) when estrous cycle stage was not taken into account.

### Ovariectomy induces an increase in body weight in both conventional and germ-free mice

**Figure 2:**
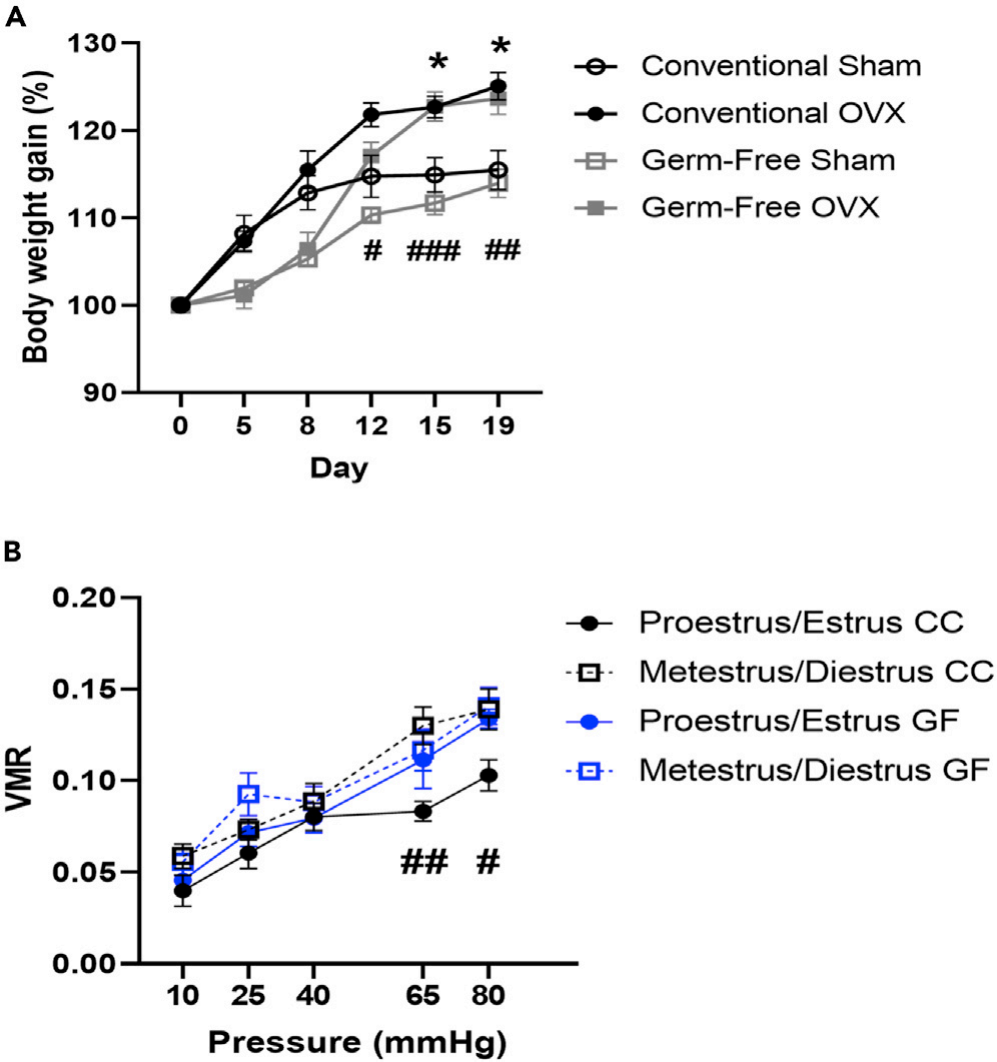
Ovariectomy induced an increase in body weight in both conventional and germ-free mice and estrous cycle modulation of visceral pain was driven by microbiota-dependent mechanisms (A) Ovariectomy (OVX) induced an increase in body weight in female mice regardless of microbiota status. ∗p ≤ 0.05 for conventionally colonized ovariectomized versus sham group, #p ≤ 0.05, ##p ≤ 0.01, ###p ≤ 0.001 for germ-free ovariectomized versus sham group. n = 7–12 per group. Data presented as Mean ± SEM. (B) Estrous Cycle modulates visceral pain perception in conventional animals only. #p ≤ 0.05, ##p ≤ 0.01 conventionally colonized proestrus/estrus versus metestrus/diestrus group. n = 8–14 per group. Data presented as Mean ± SEM. VMR, visceromotor response; GF, germ-free; CC, conventionally colonised.

Here, we observed that OVX induced an increase in body weight in both conventional and GF mice as reported previously (Ding et al., 2017) as found by a repeated measures 2-way ANOVA by a main effect of time (F(5,175) = 235.8, p< 0.001), of OVX with respect to time (F(5,175) = 21.5, p < 0.001), of microbiota status with respect to time (F(5,175) = 9.9, p < 0.001) but not of microbiota status∗OVX with respect to time (F(5,175) = 0.6, p = 0.7) (Figure 2A). Significant main effects of OVX (F(1,35) = 10.6, p = 0.003) and of microbiota status (F(1,35) = 7.1, p = 0.012) but not of a microbiota status∗OVX interaction (F(1,35) = 0.01, p = 0.921) on body weight gain were also noted (Figure 2A).

Further analysis using Bonferroni post hoc revealed specific differences in body weight between CC-Sham and CC-OVX groups on day 15 (p = 0.043), and day 19 (p = 0.023), and between GF-Sham and GF-OVX groups on day 12 (p = 0.047), day 15 (p < 0.001), and day 19 (p = 0.004), whereby the OVX groups weighed more than sham controls.

### Estrous cycle modulation of visceral pain is driven by microbiota-dependent mechanisms

In conventional females, a mixed design ANOVA with pressure as the repeated measures factor and estrous cycle stage as the independent factor revealed a main effect of pressure (F(4,80) = 21, p<0.001) but not of estrous stage with respect to pressure (F(4,80) = 1.6, p=0.171). A main effect of estrous stage alone was also noted (F(1,20) = 12.4, p=0.002) (Figure 2B). Post hoc analysis using Bonferroni correction revealed significant differences between CC animals in proestrus/estrus versus when in metestrus/diestrus at pressures of 65mmHg (p = 0.004), and 80mmHg (p = 0.037), whereby the VMR to CRD was lower in conventional animals during proestrus/estrus versus when in metestrus/diestrus (Figure 2B).

No significant differences in VMR across the estrous cycle were noted in germ-free animals using a mixed design ANOVA with pressure as the repeated measures factor and estrous cycle stage as the independent factor reporting a main effect of pressure (F(4,64) = 22.5, p<0.001) but not of estrous stage with respect to pressure (F(4,64) = 0.4, p = 0.819) nor an effect of estrous stage alone (F(1,16) = 2, p = 0.176) (Figure 2B).

When data were grouped by (1) stage of estrous and (2) microbiota status, repeated measures 2-way ANOVA also revealed a main effect of pressure (F(4,144) = 43.5, p<0.001) but not of group with respect to pressure (F(12,144) = 0.8, p=0.6). A main effect of group was also noted (F(3,36) = 5.9, p = 0.002). Further analysis using Bonferroni post hoc revealed significant differences between conventional animals in proestrus/estrus versus when in metestrus/diestrus at pressures of 65mmHg (p = 0.041), whereby conventional females in proestrus/estrus displayed lower VMR to CRD versus conventional females in metestrus/diestrus (Figure 2B).

### Ovariectomy induces visceral hypersensitivity in a microbiota-dependent manner

**Figure 3:**
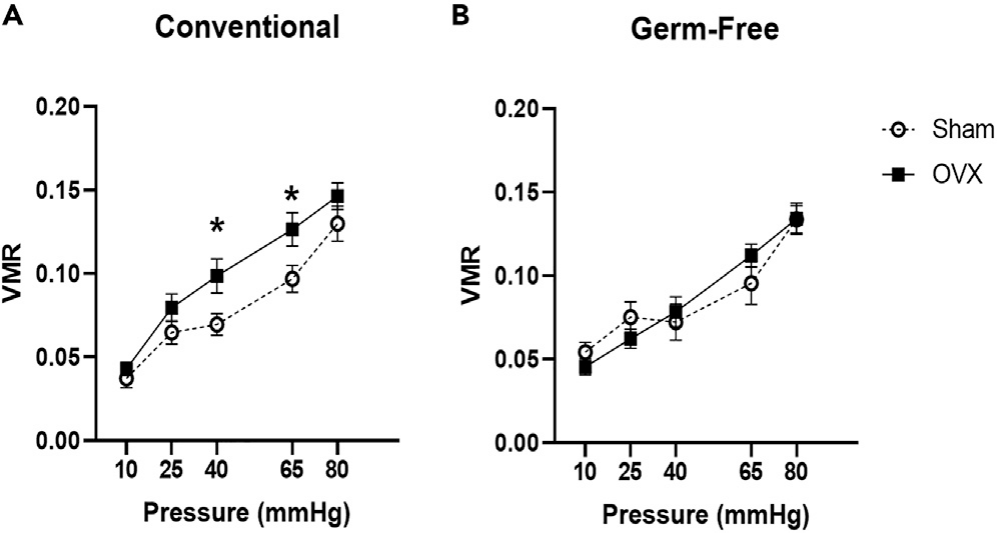
Ovariectomy induced visceral hypersensitivity in a microbiota-dependent manner (A) Ovariectomy induced visceral hypersensitivity in conventional females. ∗p ≤ 0.05 conventionally colonized ovariectomized versus sham group. (B) No effect of ovariectomy on perception of visceral pain in germ-free females. Data presented as Mean ± SEM. n = 10–12 per group. VMR, visceromotor response; OVX, ovariectomy.

A repeated measures two-way ANOVA revealed a main effect of pressure (F(4,132) = 61.3, p < 0.001) but not of microbiota status with respect to pressure (F(4,132) = 0.6, p = 0.696) nor of OVX with respect to pressure (F(4,132) = 1.1, p = 0.347) or of microbiota status∗OVX with respect to pressure (F(4,132) = 0.5, p = 0.75) on VMR to CRD. Further, no effect of microbiota status alone was noted (F(1,33) = 1.2, p = 0.281). However, main effects of OVX (F(1,33) = 5, p = 0.032) and of microbiota status∗OVX were noted (F(1,33) = 8.1, p = 0.008) (Figure 3A).

For conventional females, a mixed design ANOVA with pressure as the repeated measures factor and OVX as the independent factor revealed a main effect of pressure (F(4,68) = 37.1, p < 0.001), but not OVX with respect to pressure (F(4,68) = 1, p = 0.413). However, a main effect of OVX alone was noted (F(1,17) = 11.7, p = 0.003). Post hoc analysis using Bonferroni correction revealed significant differences in the VMR to CRD between CC-Sham and CC-OVX animals at pressures of 40mmHg (p = 0.05) and 65mmHg (p = 0.02), whereby the VMR to CRD was higher in ovariectomized animals.

A mixed design ANOVA with pressure as the repeated measures factor and OVX as the independent factor in GF females revealed a main effect of pressure (F(4,64) = 25.1, p < 0.001) but not of OVX with respect to pressure (F(4,64) = 0.6, p = 0.65), nor of OVX alone (F(1,16) = 0.2, p = 0.65) (Figure 3B).

### Hormone replacement with 17β-Estradiol reverses ovariectomy-induced visceral hypersensitivity in conventional females

**Figure 4:**
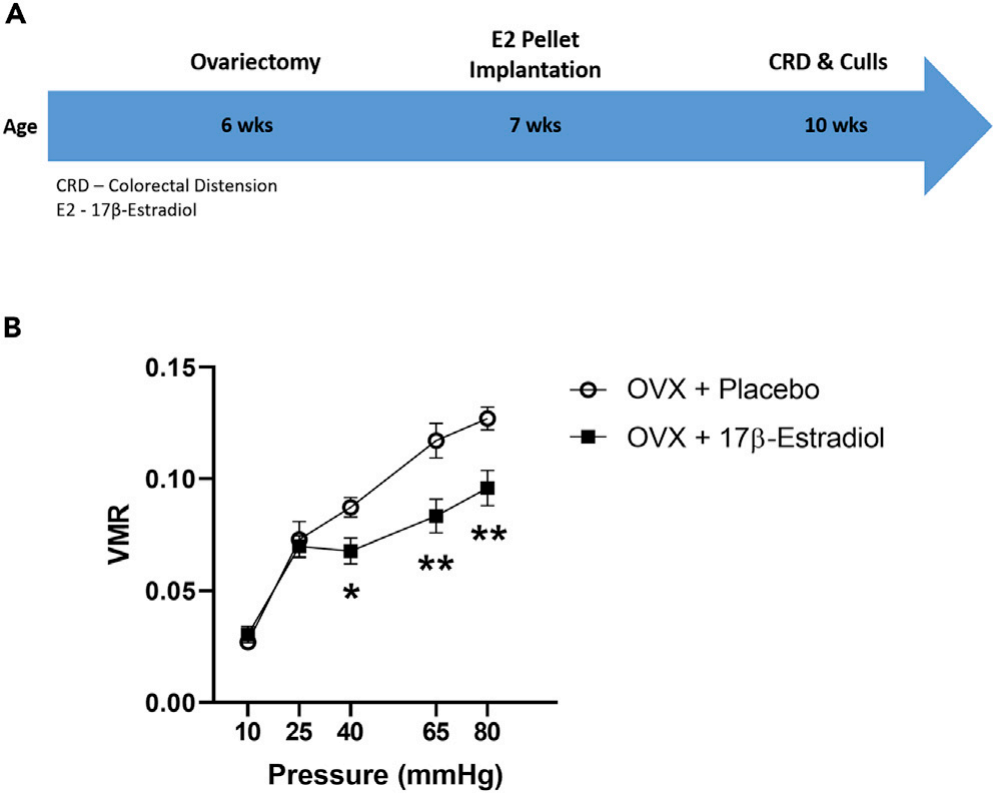
Hormone replacement with 17β-Estradiol reversed ovariectomy-induced visceral hypersensitivity in conventional females (A) Experimental timeline. (B) 17β-Estradiol reversed ovariectomy-induced visceral hypersensitivity in conventional mice. ∗p ≤ 0.05, ∗∗p ≤ 0.01 ovariectomized with estradiol versus placebo group. n = 13–14 per group. Data presented as Mean ± SEM. n = 13–14 per group. VMR, visceromotor response; OVX, ovariectomy.

To assess the role of female sex hormones in the visceral pain response, 6-week-old CC animals were ovariectomized or underwent sham surgery. At 7 weeks old, these animals underwent 17β-estradiol (E2) pellet or placebo implantation to investigate the role of E2 on perception of visceral pain (see STAR Methods). Finally, these animals underwent CRD at 10 weeks of age (see Figure 4A for experimental design).

Finally, to investigate the impact of estrogen in mediating the effects of female circulating sex hormones on the visceral pain response, ovariectomized E2 pellet-implanted animals underwent CRD. A mixed design ANOVA with pressure as the repeated measures factor and treatment as the independent factor revealed that E2 reduced the VMR to CRD by a main effect of pressure (F(4,84) = 47.5, p<0.001), of hormone replacement with respect to pressure (F(4,84) = 2.9, p=0.026) and of hormone replacement alone (F(1,21) = 20.6, p<0.001) (Figure 4B). Significant differences in the VMR to CRD were noted by post hoc analysis using Bonferroni correction at pressures of 40mmHg (p = 0.024), 65mmHg (p = 0.01), and 80mmHg (p = 0.005), whereby the VMR of E2 pellet-implanted animals was lower than that of controls (Figure 4B).

## Discussion

This study aimed to investigate the role of the microbiota and female sex hormones in OVX-induced visceral pain. We show that OVX-induced VH is dependent on the gut microbiota and that visceral pain is modulated across the estrous cycle in a microbiota-dependent manner. We also noted that OVX induced an increase in body weight regardless of microbiota status and hormone replacement with E2 ameliorated OVX-induced increases in visceral sensitivity in conventional mice. Overall, these results highlight the major regulatory role of the gut microbiota on sensation of visceral pain, as well as the potential benefit of female sex hormones in lessening the pain response to a noxious visceral stimulus.

Here, we show that contrary to our previous work in male mice (Luczynski et al., 2017), no difference was seen between control and GF female mice in the pain response to CRD when the stage of the estrous cycle was not accounted for. While there are several reports of sex differences in the functioning of the microbiota-gut-brain axis (Jaggar et al., 2020; Audet, 2019; Darch et al., 2021), we show for the first time that the gut microbiota is an important factor in sex differences in the visceral pain response. Here we show that the visceral pain response is modulated by the estrous cycle in conventionally colonized control mice but not in GF mice, further supporting the role of the gut microbiota in appropriate sensation of visceral stimuli. Specifically, we show a lesser pain response to CRD during proestrus/estrus than during the metestrus/diestrus stages, and that this stage-dependent difference in the visceral pain response is reliant upon a full complement of gut microbiota.

It has been seen previously that perception of visceral pain varies across the estrous cycle with female rats displaying a lower threshold to CRD in proestrus and estrus versus metestrus and diestrus (Moloney et al., 2016). Mechanisms behind this stage-dependent increase in VMR may lie in the heightened estrogen levels seen in proestrus which has been shown to be protective against visceral pain (Cao et al., 2012). It has also been noted that GF mice have altered sex hormone levels, and colonization of these mice increases reproductive capability, highlighting the link between the microbiota and sex hormones (Wallace et al., 2018). Furthermore, human studies have shown that women who harbor a more diverse gut microbiota display higher levels of estradiol, supporting the relationship between the gut microbiota and gonadal hormones (Shin et al., 2019). Also, women with higher serum estradiol levels had more *Bacteroidetes* and less *Firmicutes* than those with lower serum estradiol, and the genera *Slackia* and *Butyricimonas* significantly correlated with serum estradiol levels. Interestingly, the gut microbiota has been shown to significantly affect estrogen levels whereby microbial richness and diversity correlated with circulating estrogen levels (Flores et al., 2012). Hence, there is a clear association between gonadal hormones and the gut microbiota, which contributes to sex differences in the pain response.

Interestingly, in our study, OVX resulted in VH in control but not microbiota-deficient GF mice. Previous studies have shown that OVX results in visceral hyperalgesia to mechanical (von Frey) and thermal (hot plate) stimuli (Sanoja and Cervero, 2008), as well as to intracolonic capsaicin administration (Sanoja and Cervero, 2005). OVX has also been shown to cause shifts in the gut microbiota (Wang et al., 2016; Mendes et al., 2017). Specifically, OVX in rats resulted in increases in *Escherichia coli* and *Bacteroides fragilis* as well as a decrease in *Clostridium leptum*, *Faecalibacterium prausnitzii,* and *Lactobacillus*. As the gut microbiota plays a major role in visceral pain (O' Mahony et al., 2017), and GF mice are devoid of a gut microbiota, this could explain why OVX in GF animals did not result in VH. Reasoning behind a more pronounced effect of OVX on the VMR at higher distension pressures may include the observation that as distension pressure increases, so too does the VMR due to increased activation of visceral nociceptors and increased pressure in the colorectal region. Thus, the effect of OVX on visceral pain perception may be more pronounced at a higher distension pressure versus a lower pressure. This finding supports the role of the microbiota in the manifestation of VH.

Estrogen has been shown to upregulate neuronal activation in the central and peripheral nervous systems including at the level of the dorsal root ganglia in the spinal cord, GI tract, and enteric nervous system, thus exerting effects on visceral pain processing (Sun et al., 2019). The antinociceptive effects of estrogen have also been shown previously, whereby the expression of substance P, a neuropeptide related to pain, was downregulated in lumbar dorsal root ganglia neurons in OVX rats implanted with 17β-Estradiol pellets (Sarajari and Oblinger, 2010). Here, we show that OVX-induced VH was ameliorated by hormone replacement via E2 pellet implantation. Estrogen receptors ERα and ERβ are capable of modulating the visceral pain response by regulation of activity of sensory neurons (Meleine and Matricon, 2014), and estradiol has been shown to inhibit voltage-gated calcium channels of primary afferent neurons of the dorsal root ganglia in rats (Lee et al., 2002). Estrogen receptor activation has been shown previously to have analgesic effects in a visceral pain model in mice (Zielińska et al., 2017), and the gut microbiota is also capable of metabolizing estrogens via microbial-derived β-glucuronidase, which then act via ERα and ERβ (Baker et al., 2017). ERβ knockout in mice revealed that ERβ affects gut microbiota composition (Menon et al., 2013) and that sex hormones affect the gut microbiota, thus posing a potential mechanism of action of E2 on visceral pain processing. Overall, from the results herein and from existing literature, it is clear that the gut microbiota plays a major role in control of estrogenic activity which modulates the visceral pain response.

An increase in body weight following OVX has been reported previously both in rats (Sharma et al., 2017) and in mice (Ding et al., 2017). Notably, here we show that this increase in body weight after OVX is independent of gut microbiota status. This OVX-induced weight gain has previously been attributed to the loss of circulating estrogen as it is seen that exogenous addition of a phytoestrogen reduced OVX-induced weight gain (Sharma et al., 2017).

In summary, we report that visceral pain is modulated across the estrous cycle in a microbiota-dependent manner. This is, to our knowledge, the first study to demonstrate reversal of OVX-induced VH to CRD by estrogen replacement therapy by E2 pellet implantation.

### Limitations of the study

While this study provides a novel insight into the role of the microbiota in VH, further studies are needed to uncover exact molecular mechanisms behind estrous cycle modulation of visceral pain and the role the microbiota plays in this modulation.

## STAR★Methods

### Key resources table

**Table:** 

REAGENT or RESOURCE	SOURCE	IDENTIFIER
**Experimental models: organisms/strains**		

Germ-free Swiss Webster mice	Taconic, Germantown, USA.	SW-F/SW-M

**Software and algorithms**		

IBM SPSS Statistics version 27	IBM	https://www.ibm.com/support/pages/downloading-ibm-spss-statistics-27

**Other**		

17β-Estradiol pellet	Innovative Research of America, Florida, USA.	Cat # E-121
Placebo pellet	Innovative Research of America, Florida, USA.	Cat # C-111
Stainless steel reusable precision trocar	Innovative Research of America, Florida, USA.	Cat # MP-182
Autoclavable pelleted diet	Special Diets Services	Product code 801010

### Resource availability

#### Lead contact

Requests for further information or materials should be directed to the lead contact Prof. John Cryan (j.cryan@ucc.ie).

#### Materials availability

This study did not generate new unique reagents.

#### Data and code availability

• All data reported in this paper will be shared by the lead contact upon request.
• This paper does not report original code.
• Any additional information required to reanalyze the data reported in this paper is available from the lead contact upon request.

### Experimental model and subject details

For this study, naïve Swiss Webster germ-free and conventional females aged between 6 and 10 weeks old were used. All animals were group housed and littermates were randomly assigned to each experimental group. All experiments were conducted in accordance with the guidelines of European Directive 86/609/EEC and the Recommendations 2007/526/65/EC and were approved by the Animal Experimentation Ethics Committee of University College Cork. All efforts were made to reduce the number of animals used for the study and minimize animal suffering.

### Method details

#### Animals

Swiss Webster breeding pairs for germ-free (GF) and conventionally colonized (CC) groups were supplied by Taconic (Germantown, New York, USA) and first-generation female offspring were used for all experiments. GF mice were group housed in flexible film gnotobiotic isolators maintained at 21 ± 1°C with 55-60% relative humidity under a 12-hr light/dark cycle in the University College Cork GF Unit. CC mice were group housed in the standard animal facility and maintained under the same temperature, relative humidity, and light/dark cycle as the GF unit. Both GF and CC mice were age matched and fed the same autoclaved pelleted diet (Special Diets Services, product code 801010). Surgery, testing, or euthanasia occurred between 6 and 10 weeks of age. All experimenters were blinded as to the experimental groups until scoring was completed.

#### Experimental timeline

This study was performed to address two questions as set out in the objectives and the experiments were designed as follows:

(i) Visceral sensitivity assessment in CC and GF mice following ovariectomy with sham control.
(ii) Visceral sensitivity assessment in ovariectomized CC E2 pellet-implanted mice with placebo control.

#### Ovariectomy

Ovariectomy (OVX) was carried out as previously described (O'Leary et al., 2009) in 6-week-old animals under germ-free conditions. Ketamine (90mg/kg) and Xylazine (10mg/kg) (both Abbeyville Veterinary, Ireland) anesthetic mix was administered intraperitoneally to induce anesthesia for ovariectomy surgeries. An area of the dorsal surface of the animal was shaved and sterilized with 70% ethanol and a small 2cm incision was made in the center of this area. The fat pad of the left ovary was located laterally to this incision and a 0.5cm incision made through the abdominal wall to access the ovary. The ovary was gently taken out through the incision by pulling the accompanying fat and the ovary was then removed and the uterus gently replaced into the abdominal cavity. The incision site was then sutured, and the same procedure was carried out on the other side to remove the right ovary. The initial incision was then also sutured closed and cleaned with sterile saline. To control for the effect of the surgery on the study outcomes, the surgery was performed in the same manner, with the exception of the removal of the ovaries (i.e., the ovaries were gently pulled through the incision site but were immediately returned) to form the sham-OVX group. All animals received the anti-inflammatory drug carprofen (5mg/kg, s.c., Carprofen, Norbrook) 1 day after the surgery.

#### 17β-estradiol pellet implantation

17β-Estradiol (E2) pellet implantation was carried out as previously described (Ingberg et al., 2012). The pellets, 0.1mg E2/pellet (21-day release, IRA, FL, USA cat. No. E−121) or placebo (0.1mg/pellet, 21-day release, IRA, FL, USA cat. No. C-111) were implanted 1 week after the ovariectomy procedure when the animals were 7 weeks old. Animals were anesthetized with isoflurane (1.5-2% for induction) and the pellet was then inserted subcutaneously into the dorsal aspect of the neck using a stainless steel reusable precision trocar with regular medical point needle (IRA, FL. USA, cat. No. MP-182). The animal was then monitored for up to 20 min following pellet insertion. The same procedure was carried out for the placebo group with a placebo pellet being implanted in place of the E2 pellet to control for the effects of the surgery.

#### Colorectal distension

Animals were removed from the germ-free facility to undergo colorectal distension (CRD) as previously described (Luczynski et al., 2017; Tramullas et al., 2012) in 9-10-week-old animals. The CRD apparatus used consisted of a barostat (Distender Series II, G and J Electronics, Toronto, ON, Canada) and a transducer amplifier (LabTrax 4, World Precision Instruments, Sarasota, FL). A custom-made polyurethane balloon (2cm length x 1cm inflated diameter) was tied over a PE60 catheter with silk 4.0. Prior to securing the balloon to the catheter, several holes were punched in the distal 20mm of the tubing with a 27-gauge needle to allow for inflation of the balloon.

Mice were lightly anesthetized with isoflurane (2% vapor in oxygen, IsoFlo, Abbot, UK) and the lubricated balloon along with a connecting catheter were inserted into the colon, 0.5cm proximal to the anus. The catheter was secured to the base of the tail with tape to prevent removal or displacement. Animals were allowed a recovery time of 10 min prior to the commencement of the CRD procedure. The balloon was connected to the barostat system and an ascending phasic distension protocol from 10 to 80mmHg consisting of three 20s pulses at each pressure with a 5-minute inter-pulse interval was used. The visceromotor response (VMR) to CRD was quantified by the pressure changes observed within the colonic distending balloon during the procedure and the average of the three consecutive pulses for each pressure were used. For each animal, pain threshold was designated as the pressure which exceeded the mean baseline activity plus three times the standard deviation. Following the visceral sensitivity assessment, the balloon was carefully removed, and the animals were sacrificed within 4 hours.

#### Identification of stages of estrous

To investigate the effect of the different stages of the estrous cycle on visceral pain perception, vaginal cytology was used. A vaginal swab using a saline-soaked cotton swab (saline was used to help preserve the morphology of cells taken as water may disturb the structure of the cells, potentially confounding the interpretation of the stage of estrous (Cora et al., 2015)) was collected by gentle insertion of the cotton swab into the vaginal canal of the restrained animals. The cotton swab was slowly and gently rotated against the vaginal wall and then removed. Cells collected during the swab were transferred to a dry glass slide and imaged under a light microscope. The stage of the estrous cycle was determined based on the type and shape of the cells present (Caligioni, 2009). For the purpose of this study, the stages of estrous were grouped as follows; Proestrus/Estrus and Metestrus/Diestrus due to previous reports of heightened visceral sensitivity to tactile stimulation when in Metestrus/Diestrus versus Proestrus/Estrus (Gonzalez and Carrasquillo, 2019; Giamberardino et al., 1997).

### Quantification and statistical analysis

All data were assessed for normality using the Shapiro-Wilk test and Levene’s test for equality of variances. Normally distributed data were analyzed using repeated measures two-way ANOVA or mixed deign ANOVA followed by post hoc testing using Bonferroni correction. A p-value of 0.05 was set as the threshold of statistical significance and all data are presented as Mean ± SEM. Significant outliers identified in the analysis were excluded. All statistical testing was performed using SPSS version 27 (IBM Statistics). Detailed statistical reporting is listed throughout the results section under the relevant subheadings and in the figure legends. The number of animals used per experiment are as follows with n representing the individual animal:

• To investigate the role of the microbiota in the visceral pain response, 2 groups were generated: CC and GF (n = 10 per group).
• To analyze the impact of the estrous cycle on the visceral pain response, 4 groups were used: CC (P/E), CC (M/D), GF (P/E), GF (M/D) (n=8, 14, 9, 10 per group respectively).
• For investigation into the effects of female sex hormones on visceral pain perception, 2 experiments were run: Firstly using 4 groups to observe the effect of cessation of production of estrogen: CC-Sham, CC-OVX, GF-Sham, GF-OVX (n=12, 10, 10, 12 per group respectively) and secondly to investigate hormone replacement with E2: CC-OVX-Placebo, CC-OVX-E2 (n=13, 14 per group respectively).
